# Triple-Band Single-Layer Rectenna for Outdoor RF Energy Harvesting Applications

**DOI:** 10.3390/s21103460

**Published:** 2021-05-16

**Authors:** Achilles D. Boursianis, Maria S. Papadopoulou, Stavros Koulouridis, Paolo Rocca, Apostolos Georgiadis, Manos M. Tentzeris, Sotirios K. Goudos

**Affiliations:** 1 ELEDIA Research Center, ELEDIA@AUTH, School of Physics, Aristotle University of Thessaloniki, 54 124 Thessaloniki, Greece; bachi@physics.auth.gr (A.D.B.); mpapa@physics.auth.gr (M.S.P.); 2 Electrical and Computer Engineering Department, University of Patras, 26 504 Patras, Greece; koulouridis@upatras.gr; 3 ELEDIA@UniTN—DISI, University of Trento, 38 123 Trento, Italy; paolo.rocca@unitn.it; 4 School of Engineering and Physical Sciences, Heriot-Watt University, Edinburgh EH14 4AS, UK; apostolos.georgiadis@ieee.org; 5 The School of Electrical and Computer Engineering, Georgia Institute of Technology, Atlanta, GA 30332-0250, USA; etentze@ece.gatech.edu

**Keywords:** RF energy harvesting, rectenna, moth search algorithm, antenna optimization, RF-to-DC rectifier, triple-band operation, Greinacher voltage doubler, impedance matching

## Abstract

A triple-band single-layer rectenna for outdoor RF energy applications is introduced in this paper. The proposed rectenna operates in the frequency bands of LoRa, GSM-1800, and UMTS-2100 networks. To obtain a triple-band operation, a modified E-shaped patch antenna is used. The receiving module (antenna) of the rectenna system is optimized in terms of its reflection coefficient to match the RF-to-DC rectifier. The final geometry of the proposed antenna is derived by the application of the Moth Search Algorithm and a commercial electromagnetic solver. The impedance matching network of the proposed system is obtained based on a three-step process, including the minimization of the reflection coefficient versus frequency, as well as the minimization of the reflection coefficient variations and the maximization of the DC output voltage versus RF input power. The proposed RF-to-DC rectifier is designed based on the Greinacher topology. The designed rectenna is fabricated on a single layer of FR-4 substrate. Measured results show that our proposed rectenna can harvest RF energy from outdoor (ambient and dedicated) sources with an efficiency of greater than 52%.

## 1. Introduction

At present, wireless sensor network devices that use modern technology, such as the Internet of Things (IoT) [1], require less and less power for their operation [2,3]. To extend the use of these devices, i.e., to reduce their battery replacement during wireless network operation, several techniques have been developed. Radio Frequency (RF) Energy Harvesting (EH) is one of these techniques, which is mostly based on ambient radio wave sources in wireless network systems [4,5]. Although RF EH technology was introduced to the research community in recent years, noteworthy work is already reported in the literature.

The microstrip patch antenna has been widely used as an RF energy harvester in various applications and for different frequency bands of operation [6,7,8,9]. It exhibits several comparative advantages, such as ease of fabrication, relatively low cost, small physical size compared to the wavelength of the operating frequency, and medium complexity [10,11,12]. Therefore, it has been established as an attractive technique for RF EH applications [13]. The key performance numbers of an antenna to harvest satisfactory values of energy from the environment include the reflection coefficient ($S_{11}$ parameter) of the system, the gain, the half-power beamwidth (HPBW), and the efficiency [12,13]. However, the multiband operation of the antenna module in an RF energy harvesting system is often a challenging and complex task to address. The “trial and error” method is in most cases inadequate to provide feasible solutions. Therefore, the optimization method for the design of an antenna as an RF energy harvester is a promising approach.

Several studies were published in the literature involving the design of a triple-band harvesting system, e.g., a triple-band rectenna (antenna + rectifier). The authors in [14] designed an L-probe microstrip patch rectenna that was operating in the frequency bands of GSM-900, GSM-1800, and UMTS-2100. They reported an efficiency of 40% and an output voltage of 600 mV when the input power density was 500 µW/m^2^. A triple-band differential rectenna that was operating in the frequency bands of UMTS-2100, WLAN/Wi-Fi 2.4 GHz, and WiMAX was presented in [15]. A peak gain of 9.2 dBi and a maximum power conversion efficiency (PCE) of 53% at 2 GHz was achieved. The authors in [16] introduced an ultra-lightweight multiband rectenna fabricated on a paper substrate that was operating in the recently LTE bands (0.79–0.96 GHz, 1.71–2.1 GHz, 2.5–2.69 GHz). Their proposed rectifier exhibited a PCE between 11% and 30% for an input power of −15 dBm. A triple-band corrugated patch antenna integrated with a rectifier was introduced in [17]. The proposed rectenna was operating in the WLAN (Wi-Fi 2.4 GHz, Wi-Fi 5 GHz) and C frequency bands. Maximum efficiency of 54% was obtained in the frequency band of Wi-Fi 2.4 GHz. The authors in [18] designed a dual-polarized multiband rectenna that was operating in the C-band frequency range, and specifically in the 5.42 GHz, 6.9 GHz, and 7.61 GHz frequency bands. They reported a maximum PCE of 84% at the frequency of 5.76 GHz. A triple-band multibeam RF energy harvesting system that is using hybrid combining was proposed in [19]. The authors, by hybrid combining of a 16-port antenna, achieved high gain values up to 11 dBi. By applying a multi-stub impedance matching technique, they demonstrated that the proposed RF EH system can provide a PCE greater than 40%. A multi-band rectenna for RF energy harvesting at the frequency bands of 900 MHz, 1.9 GHz, and 2.4 GHz is developed in [20]. The authors reported that the proposed system can harvest up to 6.6 times more power than the single 900 MHz frequency band. Finally, a multi-band rectenna operating in the frequency bands of 840 MHz, 1.86 GHz, 2.1 GHz, and 2.45 GHz was presented in [21]. The authors designed and fabricated a modified bow-tie antenna as the RF harvester of the proposed rectenna. The reported peak efficiencies were 30%, 22%, 33%, and 16.5% at the frequencies of 840 MHz, 1.86 GHz, 2.1 GHz, and 2.45 GHz, accordingly.

Moreover, there are a few works that present an independent module (antenna or impedance matching network (IMN) and rectifier) that is suitable for RF EH systems. The authors in [22] presented a triple-band fractal antenna that was operating in the frequency bands of GSM-900, GSM-1800, and UMTS-2100 for ambient RF energy harvesting applications. They reported a maximum gain of 4.7 dBi at the frequency band of GSM-1800 for the 3D manufactured antenna. The authors in [23] designed and fabricated a triple-band IMN along with an RF-to-DC rectifier that was operating in the frequency bands of LoRa (Long Range) and mobile communication (GSM-1800, UMTS-2100) networks. They reported a maximum PCE of 71.5% for the GSM-1800 frequency band. A highly efficient and low threshold triple-band rectifier was developed in [24]. The proposed rectifier was harvesting energy from ambient RF signals at GSM-1800, Wi-Fi 2.4 GHz, and Wi-Fi 5 GHz frequency bands. The maximum conversion efficiency was 67.4% for the GSM-1800 frequency band and input power of −3 dBm. The authors in [25] presented a triple-band rectifier that was operating in the frequency bands of GSM-900, GSM-1800, and Wi-Fi 2.4 GHz. They reported a measured RF-to-DC conversion efficiency of 52% for an input power of 0 dBm and the frequency band of GSM-900. Finally, a triple-band rectifying circuit for wireless-body sensor network applications was introduced in [26]. The authors designed and fabricated an IMN and an RF-to-DC rectifier operating in the mobile communication bands of GSM-900, GSM-1800, and UMTS-2100 and achieving a conversion efficiency of 31.2% for an RF power input of −20 dBm.

Stochastic algorithms are classified into heuristics and meta-heuristics. The latter ones, due to their trade-off mechanisms of randomization and local search which they are equipped, usually achieve a better performance score [27]. Moth Search (MS) is a newly introduced metaheuristic algorithm for global optimization problems [28]. It is a bio-inspired algorithm that is based on two distinctive movement features of moths in nature; the phototaxis, i.e., a movement of a living organism (mostly referred to flying insects) towards to or away from a source of light, and the Lévy flight, i.e., a random path in which the step-lengths have a probability distribution whose tails are not exponentially bounded.

In this work, we design and fabricate a novel triple-band single-layer rectenna for outdoor RF energy harvesting applications. The proposed rectenna can harvest RF energy both from ambient and dedicated sources, due to its tuning operation in the frequency bands of LoRa and mobile communication (GSM-1800 and UMTS-2100) networks. It comprises a modified E-shaped patch antenna that is designed on a single layer of FR-4 substrate, providing low-cost fabrication prototyping. The triple-band operation of the antenna -module has been achieved by the combination of the MS optimization algorithm and a commercial high-frequency electromagnetic simulation software.

The main contributions of this paper are summarized as follows:

- Use of the MS algorithm (MSA) to obtain an optimal solution for the antenna module of an electromagnetic radiation harvesting system.
- Performance improvement of the IMN based on a three-step process that includes the minimization of the reflection coefficient (stopping criterion of −20 dB), the minimization of the $S_{11}$ magnitude variations at the frequencies of operation over an RF input power range of 20 dB (−10 dBm to 10 dBm), and the maximization of the provided DC output voltage at the same range of RF input power.

The RF-to-DC rectifier of the proposed rectenna is based on the Greinacher topology. To the best of the authors’ knowledge, this is the first time that (a) a triple-band rectenna is designed to operate in the frequency bands of LoRa, GSM-1800, and UMTS-2100 networks, (b) the MSA is applied to obtain an optimal solution in a real problem of application in electromagnetics, and (c) a three-step process is applied to obtain a feasible solution for the IMN of the proposed rectenna.

This work is an extended version of the preliminary study published in [29]. The rest of the paper is structured as follows. Section 2 describes the mathematical model of the MSA, the optimization process of the proposed antenna, the computation process of the proposed RF-to-DC rectifier, and the rectenna prototype fabrication. Section 3 depicts the experimental setup that has been used to perform the measurements, as well as the main results of the fabricated rectenna (reflection coefficient, radiation pattern, gain, and PCE). Finally, our concluding remarks are outlined in Section 4.

## 2. Materials and Methods

### 2.1. Moth Search Algorithm Description

Moths are a group of insects that include all the species in the order of Lepidoptera, but butterflies. Many species of moths, especially their caterpillars, are significant agricultural pests in many areas around the world. The two most important features of moths that characterize their motion in the natural environment are phototaxis and Lévy flight. Phototaxis is the tendency of a living organism to fly towards or away from a source of light. Although the cause of the phototaxis is still unknown, one of the strong hypotheses assumes that moths fly to a source light through a spiral trajectory to maintain a fixed angle to the celestial light. Lévy flight is one of the most important flight trajectories in natural environments that is adopted by many species. It is a random path in which the step size is subject to Lévy distribution, i.e., a probability distribution whose tails are not exponentially bounded. The MSA adopts these two features to model a nature-inspired swarm intelligence (SI) meta-heuristic technique that can be applied in various global optimization problems [28].

To describe the optimization process of MSA, let us consider as NPop, the population of moths, which is divided into two equal sub-populations, $NPop^{A}$ and $NPop^{B}$. Each *i* moth (*i* is the population index of the moths, i.e., i=1,2,…NPop) is ranked based on the score of its objective function. This ranking score is used to classify each moth into one of the two sub-populations. Because the *i* moth with the best position, e.g., $m_{\mathrm{best}}^{g}$ (*g* is the generation index of the moths, i.e., g=1,2,…MaxGen), will be the one that is closest to the source light, we can easily derive that the moths that are classified to $NPop^{A}$ sub-population are closer to the light source, and the moths that are classified to $NPop^{B}$ sub-population are faraway from the light source.

Based on the previous classification, the position of the *i*-th moth at the *g*-th generation in $NPop^{A}$ sub-population (Lévy flight) is expressed by

(1) $$m_{i}^{g+1}=m_{i}^{g}+f_{\mathrm{sc}}L\left(x\right)$$

where $m_{i}^{g}$ and $m_{i}^{g+1}$ are the current and the next position at generation *g*, $f_{\mathrm{sc}}$ is a scale factor associated with the optimization problem, and L(x) is the Lévy distribution defined by

(2) $$L\left(x\right)=\frac{\left(index-1\right)\Gamma \left(index-1\right)sin\left(\frac{\pi (index-1)}{2}\right)}{\pi x^{index}}$$

where index∈(1,3] is an index number, Γ is the gamma function, *x* is a positive number, and $f_{\mathrm{sc}}$ is a scale factor given by

(3) $$f_{\mathrm{sc}}=\frac{step_{\max}}{g^{2}}$$

where $step_{\max}$ is the maximum step size of the random path that is subject to Lévy distribution.

Accordingly, the the position of the *i*-th moth at *g*-th generation in $NPop^{B}$ sub-population (straight flight) is expressed by

(4) $$\begin{array}{c} m_{i,j}^{g+1}=\left\{\begin{array}{c} \left(m_{i,j}^{g}+f_{\mathrm{ac}}\times \left(m_{\mathrm{best},j}^{g}-m_{i,j}^{g}\right)\right),\;rand\geq 0.5\\ \left(m_{i,j}^{g}+\frac{1}{f_{\mathrm{ac}}}\times \left(m_{\mathrm{best},j}^{g}-m_{i,j}^{g}\right)\right),\;rand<0.5 \end{array}\right. \end{array}$$

where $m_{\mathrm{best},j}^{g}$ is the best solution (the moth member of the population with the best position) at *g*-th generation, *j* is the decision variables index of the optimization problem, i.e., j=1,2,…MaxVar, and $f_{\mathrm{ac}}$ is an acceleration factor associated with the optimization problem, which is given by

(5) $$f_{\mathrm{ac}}=\frac{\sqrt{5}-1}{2}$$

In (4), the position vector of the g+1 generation is controlled by a random number rand∈[0,1], thus with a probability of 50% to be updated by one of the two equation parts. Algorithm 1 summarizes the pseudo-code of the optimization process described by the MSA.

The time complexity of the MS algorithm at each iteration can be expressed as O(NPop×D+NPop×F), where *NPop* is the population size, *D* is the number of decision variables, and *F* is the time complexity of the given optimization function.
**Algorithm 1** Pseudo-code of the Moth Search Algorithm. 1:Define the population number of moths NPop, the sub-populations $NPop^{A}$ and $NPop^{B}$, the maximum number of generations MaxGen, and the number of decision variables MaxVar 2:Define the maximum step size of random path in Lévy distribution $step_{\max}$ = 1.0 3:Set *g* = 1 4:**for** (*g* = 1; g++; g≤MaxGen) **do** 5: Initialize the position $m_{i,j}^{g}$, the best position $m_{\mathrm{best},j}^{g}$ and the fitness value $OF(m_{i,j}^{g})$ of each *i*-th moth in every *j*-th decision variable at *g*-th generation 6: ——————————Lévy flight—————————— 7: Set iA = 1 and index = 1.5 8: **for** (iA = 1; iA++; $iA\leq NPop^{A}$) **do** 9:    Compute the scale factor $f_{\mathrm{sc}}$ using (3)10:  Compute the Lévy distribution parameter value using (2)11:  Compute the position of each moth using (1)12: **end for**13: ——————————Straight flight——————————14: Set iB = 115: **for** (iB = 1; iB++; $iB\leq NPop^{B}$) **do**16:  Set *j* = 117:  **for** (*j* = 1; j++; j≤MaxVar) **do**18:   **if** (rand≥0.5) **then**19:    Compute the position of each moth using (4a)20:   **else**21:    Compute the acceleration factor using (5)22:    Compute the position of each moth using (4b)23:   **end if**24:  **end for**25: **end for**26: Compute the updated fitness value $OF(m_{i,j}^{g})$ for each moth27: Update the moth with the best position $m_{\mathrm{best},j}^{g}$ based on the fitness value28:**end for**

### 2.2. MSA Performance Evaluation

The performance evaluation of the MSA is carried out by using seven popular meta-heuristic algorithms, namely the Particle Swarm Optimization (PSO) [30], the Differential Evolution (DE) [31], the Biogeography Based Optimization (BBO) [32], the Grey Wolf Optimizer (GWO) [33], the Artificial Bee Colony (ABC) [34], the Teaching Learning Based Optimization (TLBO) [35], and the Ant Colony Optimization (ACO) [36]. To evaluate the performance of MSA against the previously mentioned algorithms, 10 common test functions are used ($f_{1}$: Ackley, $f_{2}$: Bukin No. 6, $f_{3}$: Levy No. 13, $f_{4}$: Schaffer No. 2, $f_{5}$: Shubert, $f_{6}$: Perm, $f_{7}$: Sphere, $f_{8}$: Sum of Different Powers, $f_{9}$: Booth, and $f_{10}$: Hartmann 3D). Details of the selected test functions can be found in Appendix A. To carry out the performance evaluation, the following parameters for the selected algorithms are considered:

- Number of independent trials: 100
- Number of iterations: 1000
- Population size: 100
- Number of decision variables: 30
- Bounds of decision variables: [−10 10]

Table 1 summarizes the performance results (mean values of the computed fitness function for the 100 independent trials) of the MSA against 7 popular meta-heuristic algorithms. From the presented results we can conclude that the MS algorithm achieves the best score in 7 out of 10 test functions, with the second-best score by the TLBO algorithm. To better evaluate the performance results, the non-parametric Friedman test is applied, with its results summarized in Table 2. Once again, from the presented results, we can conclude that the MS algorithm achieves the best mean ranking, with the second and the third-best score by TLBO and PSO, accordingly.

### 2.3. Triple-Band Single-Layer Rectenna

**Figure 1:**
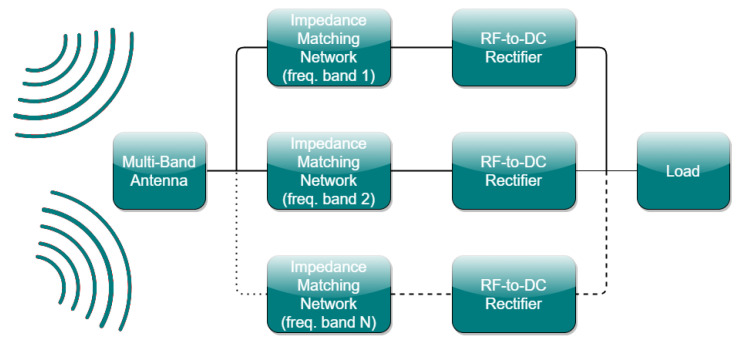
Typical multiband rectenna block diagram.

The word “rectenna” originates from the combination of the terms rectifier and antenna. Thus, in the research field of electromagnetics, a rectenna refers to the system that incorporates RF EH capabilities from ambient and dedicated RF sources, as well as power conversion capabilities from the RF input to a DC output. The first feature is acquired by the use of an RF receiving module, such as an antenna operating at specific operating frequency bands of interest. The latter one is obtained by the application of a power conversion module, such as an RF-to-DC rectifier. To achieve the maximum PCE, i.e., the maximum RF power conversion to DC output voltage, the two aforementioned modules should be matched in terms of their impedance. Therefore, an IMN is often required between the antenna and the RF-to-DC rectifier to adjust the impedance of these two modules, which in most cases differs. Figure 1 illustrates a typical multiband rectenna block diagram.

#### 2.3.1. Antenna Design Procedure

To design a multiband antenna as an RF receiving module in a rectenna, suitable for RF EH applications, is, in most cases, a complex and demanding task. The analytical approach of this task is not a convenient solution, and, in many cases, will lead to poor results. Thus, in most cases, an optimization method is required. In this work, we combine the MSA as a global optimizer in real engineering problems along with a high-frequency electromagnetic solver (HFSS, © 2020 ANSYS, Inc., Canonsburg, PA 15317, USA) to obtain a feasible solution of the antenna receiving -module.

**Figure 2:**
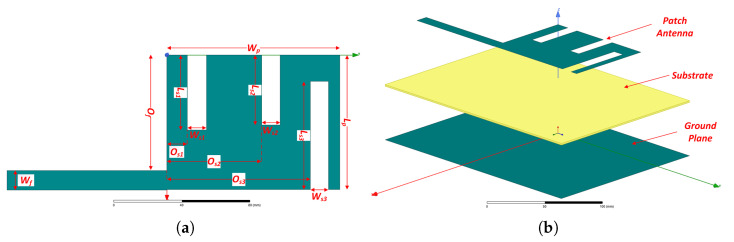
Geometry of the proposed modified E-shaped patch antenna: (**a**) top view (the decision variables of the optimization process are indicated, substrate and ground plane are omitted in this view), (**b**) expanded perspective view.

Figure 2 displays the geometry of the proposed modified E-shaped patch antenna as a receiving module in a rectenna system. It comprises a patch antenna with a modified E-shape as an RF energy harvester. The length of the microstrip line is properly selected to adjust the input impedance of the patch antenna to the characteristic impedance of 50 Ω. The antenna is placed on an FR-4 substrate (thickness = 1.6 mm, relative permittivity $\epsilon_{r}$ = 4.4, tanδ = 0.02 (Values are retrieved from the commercial solver’s database.). Beneath the substrate, a ground plane is adjusted. We should point out that boundary conditions of finite conductivity, i.e., conductivity = 5.80 × 10^7^ Siemens/m and relative permeability = 1, are adopted for the radiator, the microstrip line, and the ground plane of the proposed antenna.

If we observe the shape of the radiator that is illustrated in Figure 2a, we can conclude that the proposed modified E-shaped antenna requires 13 parameters (i.e., decision variables of the optimization problem) to describe its full geometry. Therefore, in our case, and to define these parameters, the choice of an optimization algorithm, such as the MSA, is a straightforward process. The objective of the previously described optimization problem is to minimize the reflected power of the proposed antenna, i.e., to minimize the reflection coefficient or the $S_{11}$ magnitude of the proposed antenna. The minimization of the reflected power should occur at three different frequencies that are included in the frequency bands of (a) LoRa (863–870 MHz), (b) GSM-1800 (1710–1880 MHz), and (c) UMTS-2100 (1905.1–2155.3 MHz). At each iteration, an obtained solution by the high-frequency electromagnetic simulation software is accepted and stored, if the reflection coefficient value ($S_{11}$ magnitude) is equal to or less than a predefined limit. Considering all the above, the objective function of the given optimization problem can be formulated as

(6) $$\begin{array}{c} F\left(x\right)=max\left(S_{11}^{867\mathrm{MHz}}\left(x\right),S_{11}^{1800\mathrm{MHz}}\left(x\right),S_{11}^{2100\mathrm{MHz}}\left(x\right)\right)+\Xi \times max\left(0,S_{11}^{867\mathrm{MHz}}\left(x\right)-L_{\mathrm{dB}}\right)\\ +\Xi \times max\left(0,S_{11}^{1800\mathrm{MHz}}\left(x\right)-L_{\mathrm{dB}}\right)+\Xi \times max\left(0,S_{11}^{2100\mathrm{MHz}}\left(x\right)-L_{\mathrm{dB}}\right) \end{array}$$

where

- x is the vector representing the solution (each value of the solution vector corresponds to the members of the moth population) of the proposed antenna geometry at each iteration,
- $S_{11}^{867\mathrm{MHz}}$, $S_{11}^{1800\mathrm{MHz}}$, and $S_{11}^{2100\mathrm{MHz}}$ are the values of the reflection coefficient at the solution frequencies, which fall into the desired frequency bands of LoRa, GSM-1800, and UMTS-2100,
- $L_{\mathrm{dB}}$ is the specific limit of the reflection coefficient whether a current solution of the optimization process is accepted or not ($L_{\mathrm{dB}}$ = −10 dB), and
- Ξ is a very large number that is assigned to the current solution ($S_{11}$ magnitude) of the optimization process (Ξ = 1 × 10^12^).

The MS algorithm is properly configured by applying the following parameters:

- Total population number of moths NPop: 50
- Number of sub-population $NPop^{A}$: 25
- Number of sub-population $NPop^{B}$: 25
- Number of decision variables MaxVar: 13
- Maximum number of generations MaxGen: 1000
- Number of independent trials: 10

The optimization process is applied as follows. Firstly, the parameters of the MSA are defined for each member of the moth population NPop. A set of decision variables MaxVar is selected, which defines the geometry of the proposed antenna model, for each member of the moth population. Secondly, the antenna model is parsed from the high-frequency electromagnetic solver and the reflection coefficient at the desired frequencies is computed. Thirdly, based on the output of the solver, the position of each moth at sub-populations $NPop^{A}$ and $NPop^{B}$ is also computed. Finally, the fitness function for each member of the population is evaluated and the above process is repeated until stopping criteria are met.

Table 3 lists the optimal solution (decision variables of the optimization problem) of the modified E-shaped patch antenna obtained by the optimization process using the MS algorithm. This feasible solution corresponds to the parameters of the proposed antenna that describe its geometry.

#### 2.3.2. Proposed RF-to-DC Rectifier Design

The rectifier is the key element of the rectenna system and the cornerstone of the overall design. It detects and converts RF energy to DC voltage, to power up low consumption electronic devices or charge a battery cell [37]. The indispensable rectifying elements of the circuit can be either a set of diodes or transistors. In the proposed design, we used a Schottky diode because of its low threshold voltage [38]. Specifically, the Avago’s HSMS285C (SOT-323) zero bias Schottky diode, with a low forward voltage value of $V_{F}$ = 150 mV, is used [39].

**Figure 3:**
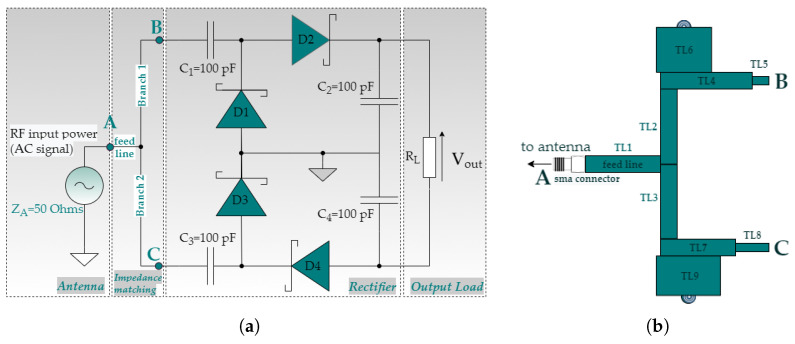
Proposed configuration of the RF-to-DC rectifier (**a**) Greinacher voltage-doubler design and (**b**) Impedance matching network design.

Figure 3 portrays the configuration of the Greinacher voltage-doubler that has been applied in our design. The voltage-doubler uses two zero bias Schottky surface mount diodes (Avago HSMS285C series), and two AVX surface mount ceramic capacitors (MLCCs) $C_{1}$ = $C_{2}$ = 100 pF. Two different impedance matching branches consisting of several conductor lines, and with appropriate width (W) and length (L), comprise the proposed IMN. This design ensures impedance matching between the rectifier and the antenna for the given frequencies, as Figure 3b depicts. For the configuration of the proposed design, we consider a standard antenna port of $Z_{A}$ = 50 Ω. Table 4 lists the optimal physical parameters of the transmission lines obtained by the S-parameter simulation controller toolbox (ADS—© Keysight Technologies 2000–2021, Santa Rosa, CA 95403–1738, USA) using the Gradient optimizer. We should also point out that, due to the variations of the input impedance of the rectifying circuit versus the RF input power, the Large-Signal S-Parameter simulation tool of the previously mentioned software is applied.

The computation process is as follows. After the selection of the circuit’s topology, as long as its peripheral elements, such as diodes, resistors, and capacitors, the Harmonic Balance method (Advanced Design System (ADS—© Keysight Technologies 2000–2021, Santa Rosa, CA 95403–1738, USA)) is applied to compute the reflection coefficient versus the frequency of the provided circuit. Also, the power conversion versus the output load is evaluated. Depending on the results, the next step includes the tuning of the physical parameters that contribute to the IMN. Finally, the efficiency versus the incident RF input signal of the overall system is computed.

#### 2.3.3. Rectenna Prototype Fabrication

**Figure 4:**
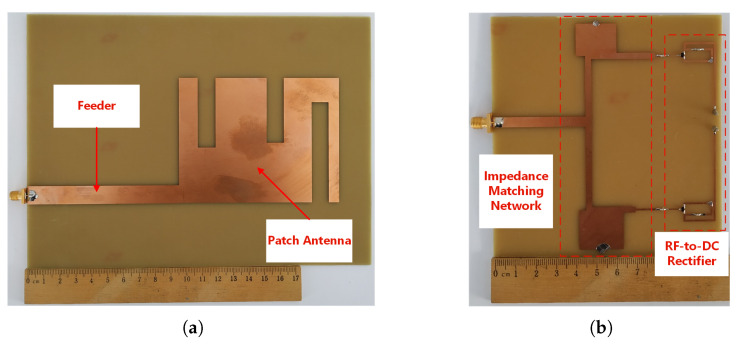
Photograph of the fabricated rectenna prototype (the ground plane is placed on the backside of the rectenna and it is omitted in the photos) (**a**) Proposed antenna and (**b**) Proposed rectifier.

Figure 4 portrays the fabricated rectenna prototype. It consists of the proposed modified E-shaped patch antenna (Figure 4a), the IMN, and the proposed rectifier (Figure 4b). The rectenna prototype is fabricated on a single layer of an FR-4 substrate. Both the rectenna and the ground plane are made of copper material (conductivity = 5.80 × 10^7^ Siemens/m, relative permeability = 1, thickness = 0.035 mm). An SMA (SubMiniature version A) connector has been soldered to the antenna and the rectifier for the experimental evaluation of the system.

## 3. Results and Discussion

### 3.1. Experimental Setup

**Figure 5:**
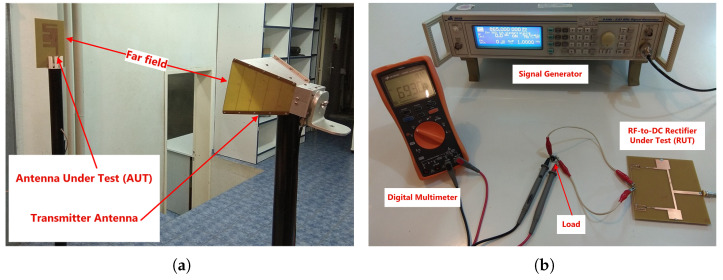
Photograph of measurement setup that was used to perform the experimental evaluation of the fabricated rectenna (**a**) Measurement setup for the proposed antenna and (**b**) Measurement setup for the proposed rectifier.

An experimental evaluation of the prototype rectenna was performed to assess its main key performance numbers. For the antenna module assessment, we have included the reflected power or the reflection coefficient versus the frequency, and the performance numbers of radiation pattern, the HPBW, and the maximum gain, at the frequencies of operation. Moreover, for the rectifier module, the DC output voltage and the PCE have been also comprised. The experimental validation of the prototype rectenna was performed in a controlled environment (see Figure 5) by using the following equipment:

- Signal Generator (© IFR Ltd. 1999), Model: IFR, Operating Frequency: 9 kHz to 2.51 GHz
- Antenna (© Keysight Technologies 2000–2021), Model: HP 11966E Double-Ridged Waveguide Horn Antenna EMCO No 3115, Operating Frequency: 1 GHz to 18 GHz (calibrated down to 750 MHz)
- Vector Network Analyzer (© 2020 Agilent Technologies, Inc.), Model: E5062A ENA-L RF Network Analyzer, Operating Frequency: 300 kHz to 3 GHz
- Spectrum Analyzer (© Keysight Technologies 2000–2021), Model: HP 8593EM EMC Analyzer, Operating Frequency: 9 kHz to 22 GHz
- Digital Multimeter (© Keysight Technologies 2000–2021), Model: U1242C RMS Digital Multimeter

The measurement process is described as follows. The vector network analyzer is used to obtain the measured reflection coefficient ($S_{11}$) for both the antenna and the RF-to-DC rectifier. For the radiation patterns and the gain of the fabricated antenna, the signal generator is used to feed the transmitter antenna (double-ridged waveguide horn antenna) at the frequencies of interest (Figure 5a). The distance between the transmitter antenna and the antenna under test (AUT) is in the far-field region. The EMC analyzer is attached to the AUT to record the measurement results. Moreover, the Friis transmission equation is used to compute the gain values of the AUT. Finally, for the PCE measurements, a signal generator is used to feed the RF-to-DC rectifier under test (RUT), with the measured DC voltage values indicated in the digital multimeter (Figure 5b).

One may notice that the fabricated antenna is experimentally evaluated in a controlled environment. Performing a set of measurements for the experimental validation of a rectifying antenna in an anechoic chamber exhibits the advantage of accuracy in the recorded values of the key performance numbers, such as the gain and the radiation pattern. The configuration of an anechoic chamber allows only the presence of the incident field in the AUT, eliminating any reflected signal and multi-path propagation. The experimental validation of a rectifying antenna in a controlled environment generally increases the measurement uncertainty, due to the presence of the reflected signals and multipath propagation. However, this feature can significantly deteriorate when the AUT, as well as the transmitting antenna, is in the far-field from any obstacle in the surrounding space.

### 3.2. Proposed Antenna Results

**Figure 6:**
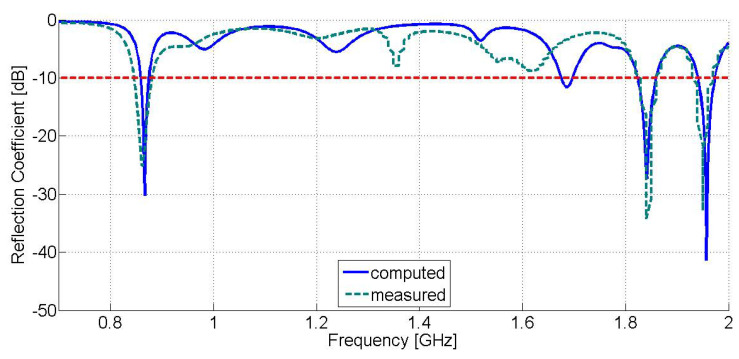
Reflection coefficient ($S_{11}$ magnitude) versus frequency of the proposed modified E-shaped patch antenna (Computed results are displayed in blue solid line, whereas measured results are displayed in dark green dash line. The red dash line represents the −10 dB limit).

Figure 6 displays the comparative results of the computed reflection coefficient against the measured one versus frequency for the proposed modified E-shaped patch antenna. From the presented graph we can derive that the receiving module of the proposed rectenna exhibits a triple-band operation (computed results: −30.26 dB at 866.4 MHz, −27.36 dB at 1.841 GHz, and −41.35 dB at 1.957 GHz, measured results: −25.2 dB at 863 MHz, −34.2 dB at 1.84 GHz, and −32.9 dB at 1.950 GHz) at frequencies that fall into the frequency bands of LoRa networks (863–870 MHz), as well as in the frequency bands of GSM-1800 (1710–1880 MHz) and UMTS-2100 (1905.1–2155.3 MHz) mobile communication networks. From the experimental results, we can conclude that the $S_{11}$ bandwidth (−10 dB limit) of the proposed antenna is about 35 MHz (LoRa: 845–880 MHz, GSM-1800: 1825–1860 MHz, UMTS-2100: 1932.5–1967.5 MHz) for each of the aforementioned frequency bands of interest, accordingly. It is worth noting that the $S_{11}$ bandwidth of the modified E-shaped patch antenna is extended to the whole frequency band of LoRa networks.

**Figure 7:**
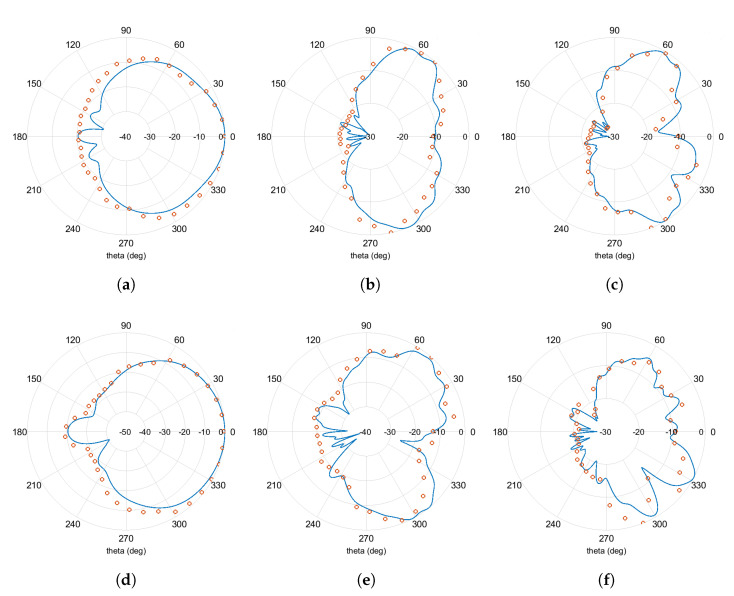
Normalized radiation pattern plots in the main planes (XZ, YZ) of the modified E-shaped patch antenna (Computed results are displayed in blue solid lines, whereas measured results are displayed in orange circular markers. Radial axis is expressed in dB.) (**a**) freq = 866.4 MHz (XZ plane), (**b**) freq = 1841 MHz (XZ plane), (**c**) freq = 1957 MHz (XZ plane), (**d**) freq = 866.4 MHz (YZ plane), (**e**) freq = 1841 MHz (YZ plane), and (**f**) freq = 1957 MHz (YZ plane).

Figure 7 illustrates the comparative results of the computed normalized radiation patterns versus the measured ones for the proposed modified E-shaped antenna in the main planes of interest (XZ: phi = 0 deg, YZ: phi = 90 deg). From the presented plots we can easily conclude that computed and measured results are in good agreement. The proposed antenna exhibits broadside beamwidth in the frequency band of LoRa networks in both main planes, and marginally acceptable beamwidth performance in the frequency bands of GSM-1800 and UMTS-2100 mobile communication networks. The maximum HPBW of the fabricated antenna reaches up to 120 deg and 97.3 deg for the XZ and YZ plane of the LoRa frequency band, accordingly.

**Figure 8:**
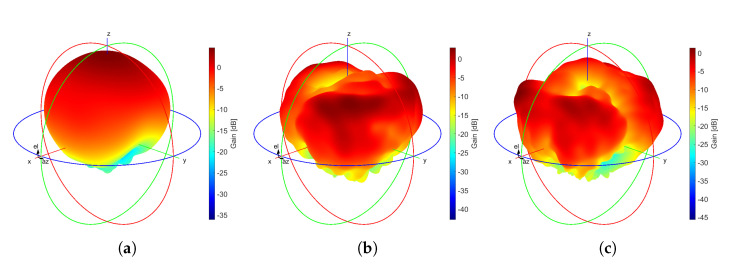
Realized gain plots (computed results) of the modified E-shaped patch antenna (color scale in dB) (**a**) freq = 866.4 MHz, (**b**) freq = 1841 MHz, and (**c**) freq = 1957 MHz.

Figure 8 portrays the computed results in a 3D polar plot of the realized gain for the modified E-shaped patch antenna. From the presented graphs, we can derive that the maximum computed values of the realized gain are 4.56 dBi, 2.95 dBi, and 1.38 dBi, for the frequency band of LoRa, GSM-1800, and UMTS-2100 networks, respectively. Following the measurement process described in Section 3.1, the maximum measured gain values of the antenna are 4.3 dBi, 2.62 dBi, and 1.14 dBi, for the previously mentioned frequency bands, accordingly. At this point, we should point out that the fabricated antenna does not exhibit high gain values. However, this is insignificant for a receiving module in a rectenna system, when it harvests the ambient energy of the surrounding environment [13,40].

### 3.3. Proposed RF-to-DC Rectifier Results

**Figure 9:**
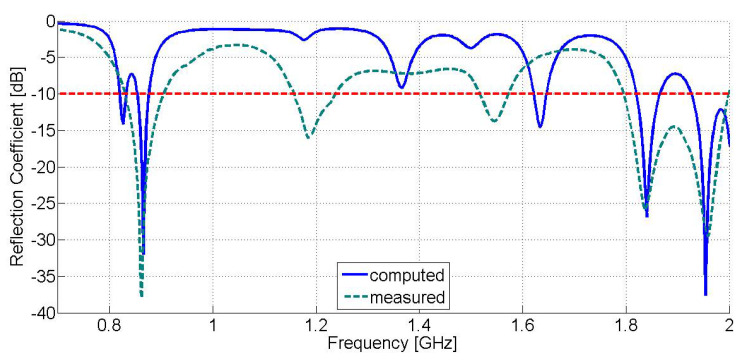
Reflection coefficient ($S_{11}$ magnitude) versus frequency of the proposed RF-to-DC rectifier at a reference level of RF input power equal to 0 dBm (Computed results are displayed in blue solid line, whereas measured results are displayed in dark green dash line. The red dash line represents the −10 dB limit).

The voltage-doubler input impedance for the triple-band design has been computed using the S-parameters simulator of the Advanced Design System (ADS). We consider a standard antenna port of 50 Ω. The size of the impedance matching circuit has been optimized and adjusted to the input impedance of the overall system. The derived values of the computed impedance are 52.43−j0.84, 53.16−j3.46, and 49.34+j1.31, at the frequencies of 865 MHz, 1840 MHz, and 1954 MHz, accordingly. Figure 9 compares the computed against the measured results versus frequency of the reflection coefficient ($S_{11}$ magnitude) for the proposed triple-band RF-to-DC rectifier at a reference level of RF input power equal to 0 dBm. From the presented graph we can derive that the triple-band rectifying circuit operates satisfactorily in the following frequency bands: (a) LoRa (863–870 MHz), (b) GSM-1800: 1710–1880 MHz, and (c) UMTS-2100: 1905.1–2155.3 MHz. The proposed rectifier shows a quite-acceptable resonance at three different frequencies which reside in the aforementioned frequency bands. The computed results of the triple-band operation are: −32 dB at 865 MHz, −26.87 dB at 1840 MHz, and −37.61 dB at 1954 MHz, whereas the measured results are: −38 at 862.5 MHz, −25.94 dB at 1837 MHz, and −29.57 dB at 1958 MHz. Taking into consideration the experimental results, we can derive that the $S_{11}$ bandwidth (−10 dB limit) of the proposed rectifier is 75 MHz and 203 MHz, for the frequency bands of LoRa and the frequency bands of GSM-1800 and UMTS-2100, accordingly.

**Figure 10:**
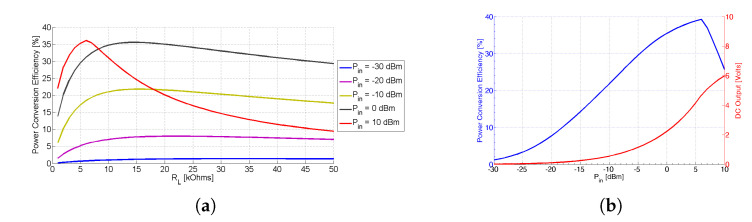
(**a**) Computed RF-to-DC power conversion efficiency versus the output load resistance for various levels of the $P_{\mathrm{in}}$ and (**b**) Total computed RF-to-DC power conversion efficiency (triple-tone) and DC output versus $P_{\mathrm{in}}$.

Figure 10a displays the computed RF-to-DC PCE as a function of the output load resistance for various levels of the input power $P_{\mathrm{in}}$. From the presented plots we can easily derive that the optimum (i.e., the load value that achieves the maximum PCE) output load is equal to 14 kΩ. The overall system RF-to-DC efficiency *n* is computed as

(7) $$n=\frac{P_{\mathrm{DC}}}{P_{\mathrm{in}}}$$

(8) $$P_{\mathrm{DC}}=\frac{V_{\mathrm{out}}^{2}}{R_{L}}$$

where $P_{\mathrm{DC}}$ is the DC output power, $P_{\mathrm{in}}$ is the RF input power, $V_{\mathrm{out}}$ is the output DC voltage, and $R_{L}$ is the output load resistance. Figure 10b portrays both the PCE and the DC output voltage versus the input power level for the proposed triple-band rectifier. The maximum efficiency is 39.5% for an input power level of 6 dBm, whereas the efficiency is greater than 20% for an RF input signal greater than −11 dBm. Also, it is worth noting that the DC output is above 0.2 V for an input power level equal to −16 dBm, whereas for RF power greater than −11 dBm, the DC output value surpasses 0.5 V.

**Figure 11:**
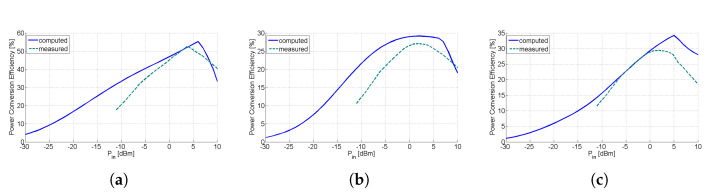
Efficiency versus RF input power level of the proposed RF-to-DC rectifier (Computed results are displayed in blue solid line, whereas measured results are displayed in dark green dash line) (**a**) freq = 866.4 MHz, (**b**) freq = 1841 MHz, and (**c**) freq = 1957 MHz.

Figure 11a demonstrates the comparative results of the computed against the measured efficiency versus the input power level at the frequency of 865 MHz (LoRa). The measured peak efficiency is 52.6% for an input power of 2.8 dBm. Also, the input power dynamic range for efficiency greater than 20% is 20 dB (−10 dBm to 10 dBm). Figure 11b illustrates the comparative results of the efficiency at the frequency of 1840 MHz (GSM-1800). The maximum measured efficiency is 27.1%. The dynamic range of the input power for efficiency greater than 20% is 16.2 dB (−6.2 dBm to 10 dBm). Finally, Figure 11c presents the comparative results of the efficiency at the frequency of 1954 MHz (UMTS-2100). From the given graph we can derive that the peak efficiency is 30% and the input power dynamic range for efficiency greater than 20% is 15 dB (−6.2 to 8.8 dBm). It is worth noting that computed and measured results of the PCE are in acceptable agreement.

### 3.4. Proposed Rectenna Performance Evaluation

Table 5 lists the comparative measured results of the proposed work against selected related publications from the literature. The key parameter numbers that are selected for comparison are the substrate of the rectenna, the frequency bands of operation, the maximum achieved gain of the antenna, the implemented technique for the IMN, the RF input power level, and the PCE, as well as the DC output voltage of the RF-to-DC rectifier. From the presented results we can conclude that our proposed rectifier exhibits competitive results against other related work. It is fabricated on a cheap substrate (FR-4) and it presents a fine-tuning operation (by evaluating both the reflection coefficient of the antenna and the RF-to-DC rectifier) at the frequency bands of interest. Moreover, it has acceptable gain values at the tuning frequencies and it adopts an IMN technique with a relatively medium complexity. Finally, the proposed rectenna achieves satisfactory PCE and high DC output voltage values, which make it a strong candidate for RF energy harvesting applications.

## 4. Conclusions

In this work, we have presented a triple-band single-layer rectenna for outdoor RF energy harvesting applications. The proposed rectenna operates satisfactorily in the frequency bands of LoRa, GSM-1800, and UMTS-2100 networks. The proposed rectenna is equipped with a modified E-shaped patch antenna. The optimal solution of the antenna is obtained by combining the MS optimization algorithm and a commercial high-frequency electromagnetic solver. The IMN of the proposed rectenna is designed based on a series of shunted and shorted stubs. The final geometry of the IMN is obtained by applying a three-step process, including the minimization of the refection coefficient values at the desired frequencies of operation, the minimization of the variations of the reflection coefficient values over an RF input power range, and the maximization of the DC output voltage over the same RF input power range. The Greinacher voltage-doubler topology is selected to harvest RF signals into DC voltage. The proposed rectenna is fabricated and evaluated in a controlled environment. Measured results of the proposed rectenna exhibit triple-band tuning operation in the previously mentioned frequency bands, broadside operation (maximum HPBW of 120 deg), acceptable gain values (maximum gain of 4.3 dBi), and maximum PCE of 52.6%. These results make it a promising candidate for various RF harvesting applications, such as IoT or wireless sensor networks. Future work includes the investigation of alternative techniques for the feeding of the antenna, the inclusion of both the antenna and the IMN in the optimization process, and the experimental assessment of the fabricated system in a real environment by harvesting RF energy from both dedicated and ambient sources.

## Figures and Tables

**Table 1:** Performance evaluation (per test function) of MSA against selected meta-heuristic algorithms (the best values are indicated in bold).

	MSA	PSO	DE	BBO	GWO	ABC	TLBO	ACO
$f_{1}$	7.957 × 10^0^	3.023 × 10^−1^	1.102 × 10^0^	2.799 × 10^−1^	1.403 × 10^−1^	3.516 × 10^0^	**8.696 × 10^−2^**	9.439 × 10^0^
$f_{2}$	**1.107 × 10^−1^**	2.421 × 10^−1^	8.639 × 10^−1^	1.600 × 10^−1^	3.065 × 10^−1^	2.484 × 10^−1^	1.758 × 10^−1^	6.826 × 10^−1^
$f_{3}$	**0.000 × 10^0^**	1.995 × 10^−3^	1.138 × 10^−2^	2.556 × 10^−3^	3.209 × 10^−3^	9.227 × 10^−4^	2.136 × 10^−3^	8.850 × 10^−3^
$f_{4}$	8.600 × 10^−5^	**9.499 × 10^−6^**	1.414 × 10^−4^	6.298 × 10^−5^	2.577 × 10^−5^	1.100 × 10^−5^	1.815 × 10^−5^	1.258 × 10^−4^
$f_{5}$	**−1.867 × 10^2^**	−1.865 × 10^2^	−1.858 × 10^2^	−1.865 × 10^2^	−1.862 × 10^2^	−1.865 × 10^2^	−1.865 × 10^2^	−1.848 × 10^2^
$f_{6}$	**4.651 × 10^49^**	8.094 × 10^50^	6.102 × 10^56^	4.509 × 10^54^	6.080 × 10^56^	2.239 × 10^57^	5.161 × 10^51^	2.283 × 10^59^
$f_{7}$	1.009 × 10^2^	1.457 × 10^0^	1.612 × 10^1^	2.443 × 10^0^	2.296 × 10^0^	3.109 × 10^1^	**9.115 × 10^−1^**	2.192 × 10^2^
$f_{8}$	**1.844 × 10^15^**	8.963 × 10^15^	3.317 × 10^18^	2.495 × 10^17^	8.900 × 10^18^	5.373 × 10^17^	7.460 × 10^15^	3.200 × 10^19^
$f_{9}$	**4.080 × 10^−4^**	1.378 × 10^−3^	2.251 × 10^−2^	3.972 × 10^−3^	4.849 × 10^−3^	5.703 × 10^−4^	2.041 × 10^−3^	1.131 × 10^−2^
$f_{10}$	**−3.854 × 10^0^**	−3.846 × 10^0^	−3.789 × 10^0^	−3.847 × 10^0^	−3.848 × 10^0^	−3.851 × 10^0^	−3.847 × 10^0^	−3.807 × 10^0^

**Table 2:** Ranking score of the MSA against 7 popular meta-heuristic algorithms performance results based on Friedman’s non-parametric test (the best values are indicated in bold).

Algorithm	MSA	PSO	DE	BBO	GWO	ABC	TLBO	ACO
Friedman test	**2.7**	3.2	6.9	4.0	4.8	4.1	2.9	7.5
Normalized Ranking	**1**	3	7	4	6	5	2	8

**Table 3:** Optimal solution (decision variables of the optimization process) of the proposed modified E-shaped patch antenna as illustrated in Figure 2a (values are expressed in mm).

$L_{p}$	$W_{p}$	$L_{s1}$	$W_{s1}$	$L_{s2}$	$W_{s2}$	$L_{s3}$	$W_{s3}$	$W_{f}$	$O_{s1}$	$O_{s2}$	$O_{s3}$	$O_{f}$
80.29	103.41	44.76	11.45	41.71	11.03	64.70	10.96	11.58	12.20	56.60	85.70	68.71

**Table 4:** Optimal physical parameters of the transmission lines included in the proposed impedance matching network as illustrated in Figure 3b (each set of values for every physical parameter is denoted as width/length and is expressed in mm).

${\mathrm{TL}}_{1}$	${\mathrm{TL}}_{2}$	${\mathrm{TL}}_{3}$	${\mathrm{TL}}_{4}$	${\mathrm{TL}}_{5}$	${\mathrm{TL}}_{6}$	${\mathrm{TL}}_{7}$	${\mathrm{TL}}_{8}$	${\mathrm{TL}}_{9}$
4.9/46	3/33.7	3/45.6	3/33	1/3	20/15.8	3/23	1/13	22/19.8

**Table 5:** Comparative measured results of the proposed triple-band rectenna against related work.

Ref.	Substrate	Freq. Bands	Max. Gain	IMN	RF Input	PCE and $V_{\mathrm{out}}$
[14]	RT/ Duroid 5880	GSM-900, GSM-1800, UMTS-2100	8.15 dBi	Shunted and radial stubs, lumped elements	−10 dBm	40% & 0.447 V @925 MHz 31% & 0.394 V @1820 MHz 25% & 0.354 V @2170 MHz
[15]	FR-4	UMTS-2100, Wi-Fi 2.4 GHz, WiMAX	9.2 dBi	Meander line, open and radial stubs	−13.5 dBm	52% & 0.160 V @2.0 GHz 25% & 0.111 V @2.5 GHz 14% & 0.083 V @3.5 GHz
[16]	paper	LTE (0.79–0.96 GHz, 1.71–2.17 GHz, 2.5–2.69 GHz)	6.0 dBi	Shunted and radial stubs, lumped elements	−10 dBm	35% & 0.32 V @900 MHz 30% & 0.30 V @1800 MHz 28% & 0.29 V @2600 MHz
[17]	FR-4	Wi-Fi 2.4 GHz, Wi-Fi 5 GHz, C-band	4.42 dBi	Shorted stubs	−10 dBm	50% & 0.28 V @2.45 GHz 45% & 0.27 V @5.05 GHz 35% & 0.24 V @4.075 GHz
[18]	FR-4	C-band (5.42 GHz, 6.9 GHz, 7.61 GHz)	7.3 dBi	Radial, shunted, and shorted stubs	5 dBm	14% & 1.152 V @5.42 GHz 15% & 1.193 V @6.90 GHz 42% & 1.996 V @7.61 GHz
This work	FR-4	LoRa, GSM-1800, UMTS-2100	4.3 dBi	Shunted and shorted stubs	−10 dBm 5 dBm	20% & 0.529 V @866.4 MHz 13% & 0.427 V @1841 MHz 13% & 0.427 V @1957 MHz 50% & 4.71 V @866.4 MHz 26% & 3.39 V @1841 MHz 28% & 3.52 V @1957 MHz

## Appendix A. Test Functions

1. Achkely Function: $f\left(x\right)=-a\times exp\left(-b\sqrt{\frac{1}{d}\sum_{i=1}^{d}x_{i}^{2}}\right)-exp\left(-b\sqrt{\frac{1}{d}\sum_{i=1}^{d}coscx_{i}}\right)+a+exp\left(1\right)$, where *d* denotes the number of dimensions, *a* = 20, *b* = 0.2, and *c* = 2π
2. Bukin Function No. 6: $f\left(x\right)=100\sqrt{|x_{2}-0.01x_{1}^{2}|}+0.01\left|x_{1}+10\right|$
3. Levy No. 13 Function: $f\left(x\right)=sin^{2}\left(3\pi x_{1}\right)+{\left(x_{1}-1\right)}^{2}\left[1+sin^{2}\left(3\pi x_{2}\right)\right]+{\left(x_{2}-1\right)}^{2}\left[1+sin^{2}\left(2\pi x_{2}\right)\right]$
4. Schaffer No. 2 Function: $f\left(x\right)=0.5+\frac{sin^{2}\left(x_{1}^{2}-x_{2}^{2}\right)-0.5}{{\left[1+0.001\left(x_{1}^{2}+x_{2}^{2}\right)\right]}^{2}}$
5. Shubert Function: $f\left(x\right)=\left(\sum_{i=1}^{5}icos\left(\left(i+1\right)x_{1}+i\right)\right)\left(\sum_{i=1}^{5}icos\left(\left(i+1\right)x_{2}+i\right)\right)$
6. Perm Function: $f\left(x\right)=\sum_{i=1}^{d}{\left(\sum_{i=1}^{d}\left(j+\beta \right)\left(x_{j}^{i}-\frac{1}{j^{i}}\right)\right)}^{2}$, where *d* denotes the number of dimensions and β is a constant number (default value is 10)
7. Sphere Function: $f\left(x\right)=\sum_{i=1}^{d}x_{i}^{2}$, where *d* denotes the number of dimensions
8. Sum of Different Powers Function: $f\left(x\right)=\sum_{i=1}^{d}{\left|x_{i}\right|}^{i+1}$, where *d* denotes the number of dimensions
9. Booth Function: $f\left(x\right)={\left(x_{1}+2x_{2}-7\right)}^{2}+{\left(x_{1}+2x_{2}-5\right)}^{2}$
10. Hartmann 3D Function: $f\left(x\right)=-\sum_{i=1}^{4}a_{i}exp\left(-\sum_{j=1}^{3}A_{ij}{\left(x_{j}-P_{ij}\right)}^{2}\right)$, where $a={\left(1,1.2,3.0,3.2\right)}^{T}$, $A=\left(\begin{array}{ccc} 3 & 10 & 30\\ 0.1 & 10 & 35\\ 3 & 10 & 30\\ 0.1 & 10 & 35 \end{array}\right)$, and $P=10^{-4}\left(\begin{array}{ccc} 3689 & 1170 & 2673\\ 4699 & 4387 & 7470\\ 1091 & 8732 & 5547\\ 381 & 5743 & 8828 \end{array}\right)$
